# Supplementary material for: An integrated system to assess marine extinctions
Source: PLoS One. 2023 Oct 26;18(10):e0293478. doi: 10.1371/journal.pone.0293478 (PMC10602268; doi:10.1371/journal.pone.0293478)
Supplement: S1 Table — (DOCX) [file pone.0293478.s001.docx]

**S1 Table.** Descriptive features of each stage/criterion of the integrated system for assessing marine extinctions (ISAME).

| Stage/Criterion | Rationale | Illustrative examples | References^†^ |
| --- | --- | --- | --- |
| I. Genuine extinction declaration | The declaration has gone through a formal peer-review process or there is a suspicion of extinction supported by evidence. | *-* | 1, 2, |

| II. Dispute over the species’ identity | Since the species is the taxonomic hierarchy level subject to extinction, species identity must not be in doubt; otherwise, declarations may result in a mistaken categorization as extinct. | periwinkle *(Littoraria flammea),* houting *(Coregonus oxyrinchus)* | , 3, 4 |
| --- | --- | --- | --- |
| III. Arithmetic score | Formulated for defining two options: there is a possibility that the case in question will be classified as extinct, or there is no possibility that the case will be classified as extinct. | *-* | - |
| III.A1. Constant sampling effort (+4) | Sampling effort to ensure with sufficient confidence that the taxon is no longer present | largetooth sawfish *(Pristis pristis)* | 1, 5 |
| III.A2. Statistical methods (extinction) plus survey effort (+4) | Upper confidence bound of any statistical estimate of extinction dates, arguing that there is a $95\%$ probability that the taxon has disappeared. In this criterion, the extinction inference can only be considered valid if there are at least extensive surveys in the species' distribution range. Likewise, to reduce the error attributable to using different statistical methods, the system is balanced by the B3 criterion (50-year threshold) and E9 criterion (statistical methods for determining persistence). | largetooth sawfish *(Pristis pristis),* Steller's sea cow *(Hydrodamalis gigas).* | 5, 6 |
| III.B1. 50-year threshold (+3) | Considered decades ago, as a criterion for determining extinctions. Although the criterion is not always generalizable to all taxonomic groups, it helps balance other criteria. The criterion, by itself, is insufficient to declare a species extinct. | Guadalupe storm-petrel *(Oceanodroma macrodactyla)* | 7, 8, 11 |
| III.C1. Severe threatening processes (+1) | Documented evidence of threatening processes, natural or human-related, that may lead to or accelerate a species extinction. The more evidence of threats there is, the stronger the correlation supporting the species' disappearance. | periwinkle *(Pristis pristis),* Steller's sea cow *(Hydrodamalis gigas).* | 5, 10, 11 |
| III.C2. Attributes to extinction (+1) | Biological and ecological attributes that correlate with extinction proneness, as well as with its causes. The more evidence of attributes there is, the stronger the correlation supporting the species' disappearance. | Steller's sea cow *(Hydrodamalis gigas),* largetooth sawfish *(Pristis pristis)* | 6, 11, 11 |
| III.D1. Sightings in the last 10 years or in three generations (+1) | Lapse of sightings to avoid prematurely declaring an extinction. A reference point of 10 years is proposed. It was calculated by averaging the more extreme times lapsed before an extinction was declared, considering the shortest possible time of the last sighting. The sample size was 161 cases of marine extinction declarations. Additionally, other widely used guidelines such as those of the IUCN have recommended waiting at least 10 years or a lapse of three generations before considering a species extinct. | long-spined urchin *(Diadema antillarum)* | 2, 12, 13 |
| III.D2. Presence outside the distribution range (-11) | The existence of new historical records outside the documented geographical distribution of a species, which casts doubt on the knowledge of the distribution. Therefore, it is necessary to focus on the sampling effort exerted in the extended range. | Boschmai's fire coral *(Millepora boschmai)* | 14 |
| III.D3. Species’ residence in dispute (-11) | Uncertainty associated with species residence may lead to mistaken categorizations as extinct. | smalltooth sawfish *(Pristis pectinate),* largetooth sawfish *(Pristis pristis),* bottlenose dolphin *(Tursiops truncatus)* | 15, 16 |
| III.E1. Statistical methods (persistence)(-1) | Upper limit of any statistical method for estimating extinction dates, arguing that there is still a probability that the taxon is present. Adding negative criteria can rule out an extinction. | smalltooth sawfish *(Pristis pectinata)* | 16 |
| III.E2. Suitability of the site (-1) | Evidence of potential habitat for the species, which implies that the species may still be present or that the species may recolonize the site. | white-tailed eagle *(Haliaeetus albicilla),*  *Osprey (Pandion haliaetus)* | 13, 15 |
| III.E3. Proximity of the species (-1) | Existence of the species near the site where the extinction occurred, which implies that the species may still be present or that the species may recolonize the site. | smalltooth sawfish *(Pristis pectinata),* barndoor skate *(Dipturus laevis)* | 15, 16 |
| IV-A. Score $<6$ | Used to categorize cases as unverified or possibly extinct based on the available evidence. | Guadalupe storm-petrel *(Oceanodroma macrodactyla)* | 11 |
| IV-B. Score ≥6 | In the face of reasonable doubt, categorizing a case as possibly extinct (instead of extinct) may prevent a mistaken declaration of extinction. | lost shark *(Carcharhinus obsolerus)* | 17 |

^†^References supporting each stage: 1. IUCN (2012), 2. IUCN (2022), , 3. Dong et al. (2015), 4. Borcherding et al. (2010), 5. Fernandez-Carvalho et al. (2014), 6. Lee et al. (2017), 7. World Conservation Union (1982), 8. Reed (1996), 9. BirdLife international (2018), 10. Crerar et al. (2014), 11. Butchart et al. (2006), 12. Dulvy et al. (2003), 13. Lessios (2016), 14. Glynn (2011), 15. del Monte-Luna et al. (2007), 16. del Monte-Luna et al. (2009). 17. White et al. (2019).
