# Supplementary material for: An integrated system to assess marine extinctions
Source: PLoS One. 2023 Oct 26;18(10):e0293478. doi: 10.1371/journal.pone.0293478 (PMC10602268; doi:10.1371/journal.pone.0293478)
Supplement: S2 Table — (DOCX) [file pone.0293478.s002.docx]

**S2 Table.** List of 20 combinations (out of a total of $2^11=2048)$ of binary variables that are deemed contentious (i.e., rare or biologically implausible) in our classification system. The scoring method described in the text is designed to unequivocally classify the remaining 2028 combinations. Status is the result of applying the ISAME procedure to each combination. Cases 1-3 must be resolved by the issue of “questionable evidence” in Stage IV-B; cases 4–20 are already implicitly resolved because Stage IV-A is unambiguously determined in these cases.

| Case | A1 | A2 | B1 | C1 | C2 | D1 | D2 | D3 | E1 | E2 | E3 | Status |
| --- | --- | --- | --- | --- | --- | --- | --- | --- | --- | --- | --- | --- |
| 1 | 0 | 1 | 0 | 1 | 1 | 0 | 0 | 0 | 0 | 0 | 0 | PE or E |
| 2 | 0 | 1 | 1 | 1 | 1 | 0 | 0 | 0 | 1 | 1 | 1 | PE or E |
| 3 | 1 | 0 | 0 | 1 | 1 | 0 | 0 | 0 | 0 | 0 | 0 | PE or E |
| 4 | 0 | 1 | 0 | 0 | 1 | 0 | 0 | 0 | 0 | 0 | 0 | PE |
| 5 | 0 | 1 | 0 | 1 | 0 | 0 | 0 | 0 | 0 | 0 | 0 | PE |
| 6 | 0 | 1 | 0 | 1 | 1 | 0 | 0 | 0 | 0 | 0 | 1 | PE |
| 7 | 0 | 1 | 0 | 1 | 1 | 0 | 0 | 0 | 0 | 1 | 0 | PE |
| 8 | 0 | 1 | 0 | 1 | 1 | 0 | 0 | 0 | 1 | 0 | 0 | PE |
| 9 | 0 | 1 | 1 | 0 | 0 | 0 | 0 | 0 | 0 | 1 | 1 | PE |
| 10 | 0 | 1 | 1 | 0 | 0 | 0 | 0 | 0 | 1 | 0 | 1 | PE |
| 11 | 0 | 1 | 1 | 0 | 0 | 0 | 0 | 0 | 1 | 1 | 0 | PE |
| 12 | 0 | 1 | 1 | 0 | 1 | 0 | 0 | 0 | 1 | 1 | 1 | PE |
| 13 | 0 | 1 | 1 | 1 | 0 | 0 | 0 | 0 | 1 | 1 | 1 | PE |
| 14 | 1 | 0 | 1 | 0 | 0 | 0 | 0 | 0 | 0 | 1 | 1 | PE |
| 15 | 1 | 0 | 1 | 0 | 0 | 0 | 0 | 0 | 1 | 0 | 1 | PE |
| 16 | 1 | 0 | 1 | 0 | 0 | 0 | 0 | 0 | 1 | 1 | 0 | PE |
| 17 | 1 | 0 | 1 | 0 | 1 | 0 | 0 | 0 | 1 | 1 | 1 | PE |
| 18 | 1 | 0 | 1 | 1 | 0 | 0 | 0 | 0 | 1 | 1 | 1 | PE |
| 19 | 1 | 1 | 0 | 0 | 0 | 0 | 0 | 0 | 1 | 1 | 1 | PE |
| 20 | 0 | 1 | 1 | 0 | 0 | 0 | 0 | 0 | 1 | 1 | 1 | PE |
